# Neuroprotective effects of dipeptidyl peptidase 4 inhibitor on Alzheimer’s disease: a narrative review

**DOI:** 10.3389/fphar.2024.1361651

**Published:** 2024-02-09

**Authors:** Xin Jiang, Ji Li, Xiaohui Yao, Hao Ding, Aihong Gu, Zhen Zhou

**Affiliations:** ^1^ Baoying People’s Hospital, Yangzhou, China; ^2^ School of Public Health and Preventive Medicine, Monash University, Melbourne, VIC, Australia

**Keywords:** Alzheimer’s disease, neurodegenerative diseases, type 2 diabetes mellitus, dipeptidyl peptidase 4, glucagon-like peptide-1

## Abstract

Insulin resistance in brain and amyloidogenesis are principal pathological features of diabetes-related cognitive decline and development of Alzheimer’s disease (AD). A growing body of evidence suggests that maintaining glucose under control in diabetic patients is beneficial for preventing AD development. Dipeptidyl peptidase 4 inhibitors (DDP4is) are a class of novel glucose-lowering medications through increasing insulin excretion and decreasing glucagon levels that have shown neuroprotective potential in recent studies. This review consolidates extant evidence from earlier and new studies investigating the association between DPP4i use, AD, and other cognitive outcomes. Beyond DPP4i’s benefits in alleviating insulin resistance and glucose-lowering, underlying mechanisms for the potential neuroprotection with DPP4i medications were categorized into the following sections: (Ferrari et al., Physiol Rev, 2021, 101, 1,047–1,081): the benefits of DPP4is on directly ameliorating the burden of β-amyloid plaques and reducing the formation of neurofibrillary tangles; DPP4i increasing the bioactivity of neuroprotective DPP4 substrates including glucagon-like peptide-1 (GLP-1), glucose-dependent insulinotropic peptide (GIP), and stromal-derived factor-1α (SDF-1α) etc.; pleiotropic effects of DPP4is on neuronal cells and intracerebral structure including anti-inflammation, anti-oxidation, and anti-apoptosis. We further revisited recently published epidemiological studies that provided supportive data to compliment preclinical evidence. Given that there remains a lack of completed randomized trials that aim at assessing the effect of DPP4is in preventing AD development and progression, this review is expected to provide a useful insight into DPP4 inhibition as a potential therapeutic target for AD prevention and treatment. The evidence is helpful for informing the rationales of future clinical research and guiding evidence-based clinical practice.

## 1 Introduction

Alzheimer’s disease (AD) is a progressive neurodegenerative disorder with insidious onset. The underlying pathologic process of AD involves the accumulation of extracellular β-amyloid (Aβ) plaques and intracellular neurofibrillary tangles (Ferrari and Sorbi, 2021). These neuropathological changes contribute to the loss of neurons and synapses, triggering progressive cognitive impairment and further leading to the development of AD. AD disproportionately affects the elderly population. Effective treatments for preventing and curing AD are still lacking. Current treatments including cholinesterase inhibitors (e.g., Donepezil, Galantamine) and N-methyl-D-aspartate receptors (e.g., Memantine) focus primarily on managing clinical symptoms and have shown no clear benefits in preventing disease progression. As per data provided by the World Health Organization, the global population of individuals aged 60 and older is estimated to increase twofold by the year 2050, reaching 2.1 billion; and the number of individuals aged 80 or older is expected to reach 426 million (World Health Organization, 2023). Demographic aging will undoubtedly lead to an exponential rise in the new cases of AD patients and its prevalence, exacerbating AD-related societal and public health burden. In 2018, Alzheimer’s Disease International estimated that there are around 50 million people worldwide living with dementia, and this number is expected to triple by 2050 (Alzheimers Disease International, 2023). The epidemiology of AD highlights the urgency of exploring an effective therapeutic approach for preventing AD from happening and slowing its progression.

**FIGURE 1:**
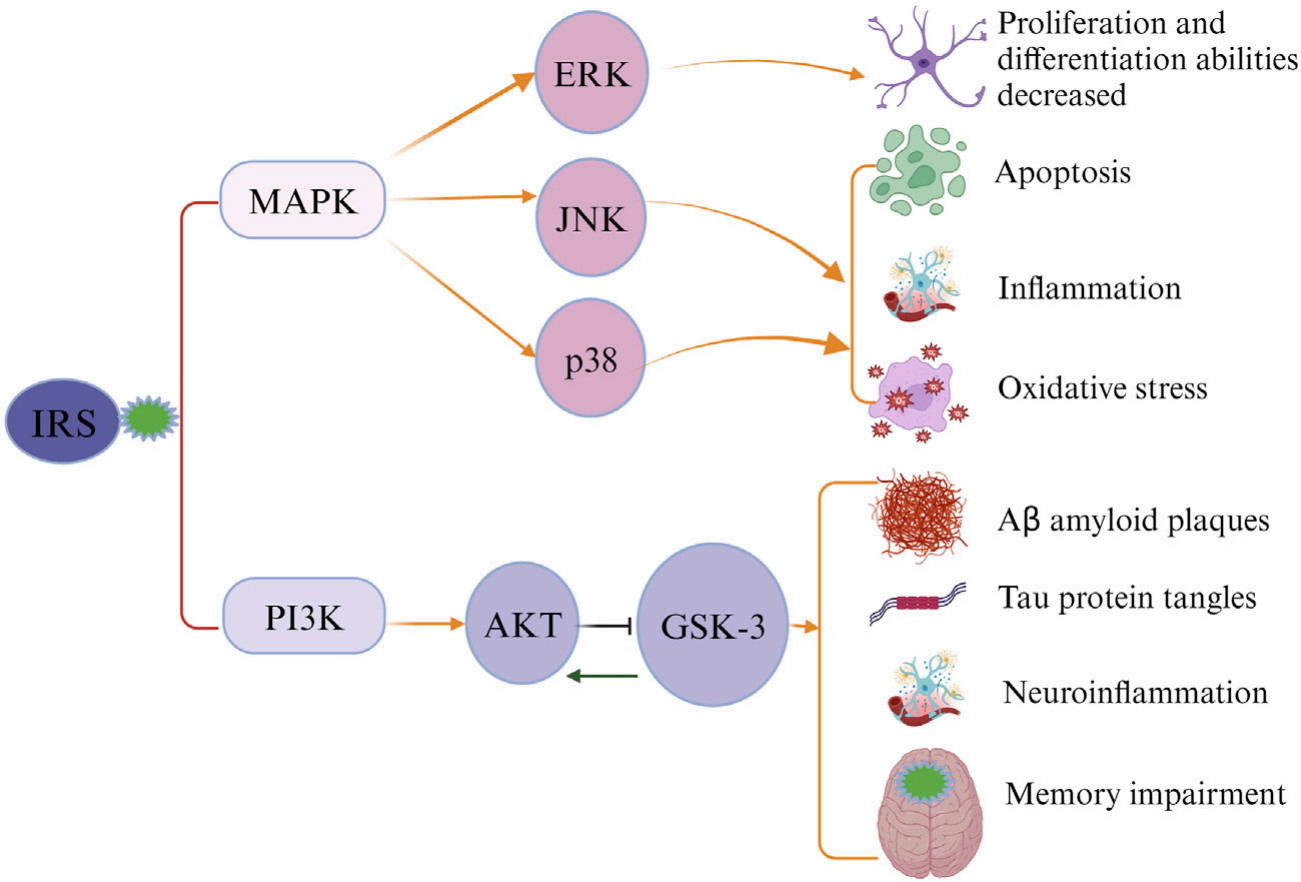
Impaired insulin signaling leads to the development of Alzheimer’s disease (AD). Insulin receptor substrate (IRS) activates the classical insulin signaling pathway. When the signaling pathway is compromised, IRS cannot activate PI3K and MAPK, causing alterations in the levels of downstream proteins and subsequently triggering the pathological characteristics of AD.

Ample evidence has suggested an association between type 2 diabetes (T2DM), cognitive decline, and AD (Talbot et al., 2012). Maintaining glucose under control in T2DM patients may serve as an effective way for preventing AD development. This hypothesis is supported by existing evidence. First, hyperglycemia was found that can substantially increase levels of Aβ protein (Yang et al., 2013; Chao et al., 2016). Second, the increased formation and accumulation of methylglyoxal through glycolytic pathways in individuals with diabetes has been linked to an increased risk of AD (Angeloni et al., 2014). Methylglyoxal, a highly reactive dicarbonyl metabolite, serves as a potent precursor for advanced glycation end products (AGEs) (Waqas et al., 2022). Both methylglyoxal and its derived AGEs are implicated in etiopathogenesis and progression of AD, inducing extensive protein cross-linking, mitochondrial dysfunction, oxidative stress, and neuronal cell death (Angeloni et al., 2014; Brings et al., 2017; Akhter et al., 2021). Studies have also found that AGEs can accumulate in neurons and astroglia, contributing the formation of neuritic amyloid plaques and neurofibrillary tangles (Srikanth et al., 2011; Fawver et al., 2012; Angeloni et al., 2014; Twarda-Clapa et al., 2022). Additionally, methylglyoxal was found that can impair the integrity of blood brain barrier (BBB), further elevating the risks of various neurodegenerative disorders, including AD and cerebrovascular diseases (Daneman and Prat, 2015; Profaci et al., 2020; Chojdak-Lukasiewicz et al., 2021; Berends et al., 2023). Third, AD is called ‘type 3’ diabetes due to the involvement of insulin resistance in its pathology (Michailidis et al., 2022). Dysfunction in brain insulin signaling is likely a pivotal factor initiating pathological changes in AD (De Felice et al., 2022). The classical insulin signaling pathway in the brain involves the activation of the PI3-K/Akt pathway by insulin receptor substrate (IRS) (Kothari et al., 2017; Salas et al., 2018; Gabbouj et al., 2019). Inhibition of this pathway can result in the blockade of downstream GSK-3, which is implicated in tau protein hyperphosphorylation, augmentation of Aβ production, neuroinflammation, and memory impairment (Chami et al., 2016; Huang et al., 2018). An alternative insulin signaling pathway, the MAPK pathway comprising ERK, JNK, and p38 kinases, was identified to be associated with cell apoptosis, neuroinflammation, and oxidative stress in the brain, contributing to the development of AD (Figure 1) (Morrison, 2012; Asih et al., 2020; Tian et al., 2023; Zhang et al., 2023).

Except for insulin resistance, common pathological features shared by T2DM and AD also include amyloid accumulation, and accumulated amylin in T2DM has a similar structural morphology as abnormal Aβ peptides in AD (Stanciu et al., 2020). Diabetes and AD have also been found to share many risk factors, which include but not limit to hyperlipidemia, metabolic syndromes, oxidative stress and inflammation, mitochondrial dysfunction, as well as genetic (e.g., amyloid precursor protein [APP] gene) and lifestyle factors (e.g., sedentary lifestyle and poor dietary patterns) (Stanciu et al., 2020; Michailidis et al., 2022). Diabetes can also cause intracerebral micro- and macro-vascular lesions to disrupt brain blood flow, contributing to an increased AD risk. An experimental study of mice models exposed to hyperglycemia condition revealed that high blood glucose levels can augment the vulnerability of endothelial cells in brain’s blood vessels to the toxicity of abnormal Aβ protein. This increased susceptibility consequently contributes to the impairment of BBB, reduced blood flow and slow clearance of Aβ protein (Carvalho et al., 2014).

Against this background, glucose-lowering medications may hold promise for repurposing as a preventive and therapeutic treatment for AD (Wang et al., 2023). A recent meta-analysis in 2022 including 229,110 participants found no evidence of protective effect of metformin on AD prevention, with of odds ratios (ORs) of 1.17 (Luo et al., 2022). Despite the unfavorable outcome of older glucose-lowering medications yielded in earlier studies, recent research has revealed the potential neuroprotective benefit associated with novel antidiabetic drugs, including sodium-glucose cotransporter-2 inhibitors (SGLT2i), dipeptidyl peptidase-4 inhibitors (DPP4is), and glucagon-like peptide-1 receptor agonists (GLP-1RAs) (Sim et al., 2021; Kopp et al., 2022; Yin et al., 2022; Sim et al., 2023). These findings are of particular interest to researchers and clinicians, and a timely review to consolidate existing evidence will be beneficial for informing current clinical practice and providing guidance for future research in more profound investigations. Given the limited number of clinical studies investigating the relationship between SGLT2i and GLP-1RA with AD, this review specifically focused on the relationship between DPP4i/gliptins and AD (Tang et al., 2023).

## 2 DPP4 inhibitor in treating T2DM

**FIGURE 2:**
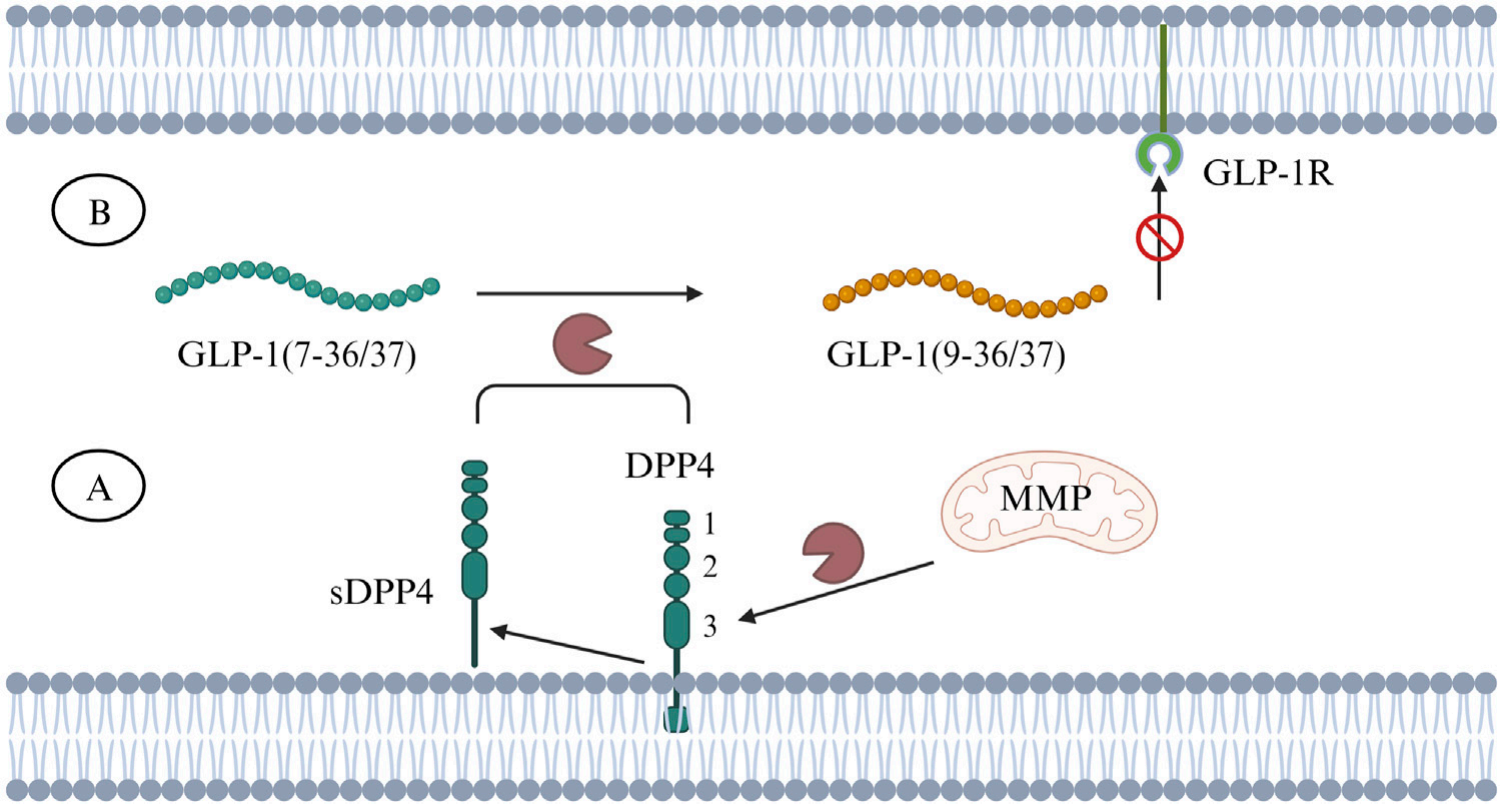
**(A)** DPP4 is a transmembrane protein and can be cleaved by MMP into sDPP4. The number 1, 2, 3 represent different regions of DPP4’s domain structure, 1. catalytic region, 2. cysteine-rich region, 3. highly glycosylated region. **(B)** Both DPP4 and sDPP4 can cleave GLP-1 (7–36/37) to inactive fragments - GLP-1 (9–36/37), making it unable to bind to GLP-1R. Abbreviations: DPP4, dipeptidyl peptidase-4; GLP-1, glucagon-like peptide-1; GLP-1R, glucagon-like peptide-1 receptor; MMP, matrix metalloproteinase; sDDP4, soluble DDP4.

DPP4 is a type Ⅱ transmembrane protein, belonging to the serine peptidase subfamily S9B, with a typical α/β hydrolase fold (Rohrborn et al., 2015). DPP4 exists in two forms: either as a membrane-anchored protein or as a soluble form (sDPP4) comprising the majority of extracellular DPP4 protein, produced through the cleavage of membrane-bound DPP4 by matrix metalloproteinase (MMP) (Rohrborn et al., 2014). sDPP4 lacks intracellular tail and transmembrane regions but retains catalytic activity (Mulvihill and Drucker, 2014). Membrane-bound DPP4 is widely expressed on the cell surface of various tissues, including intestine, liver, pancreas, kidney, spleen, lung and bone marrow, while sDPP4 is widely distributed in serum and body fluids such as saliva, cerebrospinal fluid, seminal fluid and bile (Mulvihill and Drucker, 2014; Baggio et al., 2020). Active sDPP4 in the circulation ensures that DPP4 can play a role (DPP-4-mediated proteolysis) in the extracellular environment. sDPP4 has been identified as a new adipokine contributing to most para- and endocrine effects (Rohrborn et al., 2015). In human’s brain, DPP4 was found that is expressed in thalamus, cerebral cortex, white matter, and pons (DPP4, 2024). The structure of DPP4 includes three main regions which are catalytic region, cysteine-rich region, and highly glycosylated region (Figure 2). Glucagon-like peptide-1 (GLP-1) is a peptide hormone known to maintain glucose homeostasis by enhancing glucose-dependent insulin secretion from pancreatic beta cells and suppressing the release of glucagon. DPP4 can rapidly cleave GLP-1 into inactive fragments GLP-1 (9–36/37) to prevent it from binding to GLP-1 receptors (GLP-1R) to exert an action (Figure 2). Beyond GLP-1, DPP4 can also degrade other incretin hormones, such as glucose-dependent insulinotropic polypeptide (GIP) that plays a similar role as GLP-1 on regulation of glucose and insulin (Seino et al., 2010).

DPP4i manages diabetes by stimulating insulin secretion and inhibiting glucagon secretion through elevating endogenous GLP-1 levels. Substrate-based DPP4is are drugs that bind to the active site of the enzyme, inhibiting DPP4’s activity and leading to increased levels of GLP-1. Since DPP4i agents generally do not increase the risk of hypoglycemia and is well tolerated, they have now been widely used. At least 11 different DPP4i medications have been approved for use worldwide (Deacon, 2020). Sitagliptin was the firstly approved DPP4i used on market in the United States in 2006. Vildagliptin, saxagliptin, linagliptin and alogliptin are also commonly used DPP4is as of now. DPP4 has five binding subsites including S1, S2, S1′, S2′, and S2 extensive (Arulmozhiraja et al., 2016). DPP4is interacting with S1 and S2 subsites is mandatory for them to exert their inhibitory activity, and additional interaction with S1′, S2′, or S2 extensive will substantially increase the drug’s potency (Mathur et al., 2023). Accordingly, DPP4is were grouped into different classes according to the enzyme subsites where they bind to. For example, vildagliptin and saxagliptin binding with S1 and S2 only were categorized into Class 1, alogliptin and linagliptin binding with S1′, S2′, S1 and S2 belong to Class 2, and sitagliptin, anagliptin, gemigliptin, and teneligliptin binding with S1, S2 and S2 extensive were classified as Class 3 (Arulmozhiraja et al., 2016; Gallwitz, 2019; Mathur et al., 2023).

## 3 Mechanisms underlying the potential protective benefits against AD by inhibiting DPP4 beyond glucose control

Accumulative evidence from human and experimental studies has suggested that inhibition of DPP4 may be protective against AD. A study of 1,229 Chinese adults aged 60 years old or older found that increased plasma DPP4 activity was associated with accelerated cognitive impairment and reduced MoCA score (all *p* < 0.001) (Chen et al., 2017). A significant increase in DPP4 activity was found in the brains of sporadic AD patients (Oumata et al., 2022), suggesting that increased DPP4 activity is implicated in cognitive dysfunction caused by AD. Mechanisms supporting the potential neuroprotective effects of DPP4i have been increasingly investigated and summarized as below:

### 3.1 Direct impact of DPP4i on reduction of Aβ deposition

**FIGURE 3:**
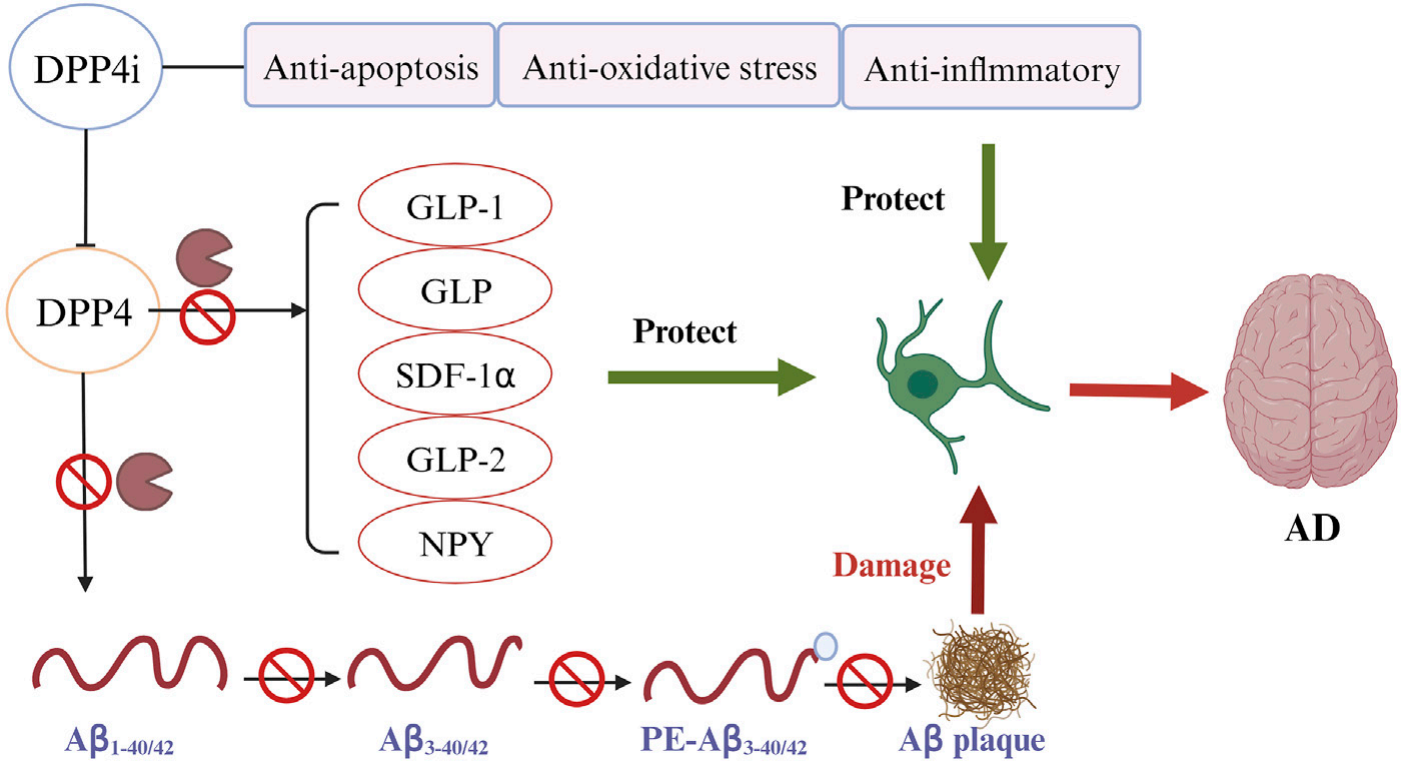
DPP4 inhibitors (DPP4i) hold potential of neuroprotective effects by inhibiting DPP4 via different mechanisms (Ferrari and Sorbi, 2021): DPP4i may be directly implicated in the prevention of Aβ plaque deposition (World Health Organization, 2023); DPP4i can increase the bioavailability of various DPP4i substrates to reduce the Aβ accumulation, tau phosphorylation and neuroinflammation (Alzheimer’s Disease International, 2023); DDP4i exhibits other beneficial properties for preventing AD, including anti-inflammation, anti-oxidation, and anti-apoptosis.

The direct impact of DPP4 on the pathological process of AD is likely explained by the DPP4’s capacity to cleave two key Aβ fragments. As observed in *in vitro* experiments, DPP4 can cleave Aβ1-42 and Aβ1-40, the crucial components of amyloid deposits in AD patients, into Aβ3-42 and Aβ3-40. Subsequently, glutamyl cyclase (GC) catalyzes the cyclization of the N-terminal glutamate of Aβ3-42 and Aβ3-40 and transforms them into non-degradable pE-Aβ3-40/42. pE-Aβ3-40/42 aggregates to form amyloid plaques and lead to the progression of AD (Antonyan et al., 2018). DPP4i can improve AD by inhibiting Aβ plaque deposition independent of GLP-1 signaling pathways (Figure 3). An experimental study found that administering DPP4i to mice can decrease the abnormal phosphorylation of Tau and neurofilaments in mice’s brain, and attenuate intracellular Aβ deposition (Chen et al., 2019). Another study in mice of AD reported similar findings that oral DPP4i administration (Linagliptin) can significantly improve incretin levels and reduce Aβ deposition, tau phosphorylation and neuroinflammation in the brain (Kosaraju et al., 2017). In agreement with these findings, an *in vitro* study of human neuronal cells found that linagliptin can restore the impaired insulin signaling caused by Aβ-induced cytotoxicity and inhibit the activation of GSK3β and hyperphosphorylation of tau by restoring insulin downstream signaling (Kornelius et al., 2015). Xue’s study on elderly T2DM patients with mild cognitive impairment revealed a significant increase in the plasma Aβ1-42/Aβ1-40 ratio in the DPP4i treatment group compared to the control group (sulfonylurea) (Xue et al., 2020). The mean values before and after DPP4i treatment were 0.39 and 0.47, respectively, while in the control group, they were 0.40 and 0.43 (*p* < 0.001). These findings suggest an improvement in Aβ burden associated with DPP4i use. In addition, a study found that inhibition of DPP4 significantly reduced the activity of β-secretase, the most important enzyme to hydrolyze APP to produce abnormal Aβ peptides (*p* < 0.01) (Huang et al., 2020).

### 3.2 DPP4i increases the bioavailability of neuroprotective DPP4 substrates

Beyond a direct effect on preventing Aβ accumulation, DPP4i may also offer benefits by increasing the expression of neuroprotective DPP4 substrates (Figure 3) (Angelopoulou and Piperi, 2018; Yin et al., 2022). GLP-1 and GIP, the best characterized DPP4 substrates, have shown their potential of neuroprotection in many studies (Holscher, 2014; Angelopoulou and Piperi, 2018; Reich and Holscher, 2022). GLP-1 and GIP can penetrate the BBB and bind to their receptors in brain tissues to exert an effect (Athauda and Foltynie, 2016; Reich and Holscher, 2022). Use of DPP4i can significantly increase the bioavailability of these two incretin hormones (Yin et al., 2022).

The neuroprotective effects of GLP-1 are mostly studied. Both human studies and studies of animal models found significantly reduced expression of GLP-1 and GLP-1R in the AD brain compared with controls without AD (Chen et al., 2019). Previous studies found extra-pancreatic effects of GLP-1 analogues which are independent of their role in glucose homeostasis. GLP-1 crosses BBB to exert neuroprotective benefits through decreasing the levels of APP and glycogen synthase kinase- 3β (GSK-3β), reducing Aβ deposition and tau phosphorylation, which are hallmarks of AD, as well as increasing insulin secretion and insulin receptor sensitivity and restoring insulin signaling pathway (Siddiqui et al., 2021). GLP-1 can also protect against neuronal degeneration by improving mitochondrial function and cellular proliferation, alleviating neuroinflammation and apoptosis (Athauda and Foltynie, 2016). Similarly, studies found that GLP-1R exists in the pyramidal neurons of hippocampus and Purkinje cells in the cerebellum. GLP-1R exerts classical type growth effects by influencing the expression of genes that are involved in cell growth and repairment. Mice overexpressing GLP-1R in hippocampus showed improved synaptic growth and cognitive function, while mice with GLP-1R knockout showed reduced synaptic plasticity and deficits in learning and memory (Du et al., 2022). The endogenous GLP-1 is quickly deactivated by the endogenous DPP4, transforming it into a metabolite that is incapable of binding to GLP-1R. While studies suggested that most DPP4is have limited ability to penetrate BBB, its inhibition of peripheral DPP4 raises the serum level of GLP-1 (Lin et al., 2023). Increasing circulated GLP-1 can penetrate BBB to exert neurocognitive benefits (Shannon, 2013). In another word, DPP4is may prevent AD development and progression by prolonging the circulating half-life of endogenous GLP-1 (Chen et al., 2019).

GIP can inhibit the apoptosis of cerebellar granule cells, and the activation of GIP receptor can promote the proliferation of neuronal progenitor cells. GIP analogues D-ala2-GIP and N-glyc-GIP have been shown to promote hippocampal synaptic plasticity and memory, while the antagonist of GIP (Pro 3-GIP) reduces hippocampal synaptic plasticity and memory (Ji et al., 2016). A novel long-acting GIP analogue DAla2GIP-Glu-PAL was shown to improve cognitive behavior, synaptic plasticity and alleviate central pathological progression in AD mice, with the underlying mechanism pertaining to the inhibition of neuroinflammation and the upregulation of cAMP-/PKA/CREB signaling pathway (Yuan et al., 2021).

Stromal-derived factor-1α (SDF-1α), also named CXCL12, is another physiological DPP4 substrate associated with neuroprotection and neurogenesis in experimental studies of AD (Chalichem et al., 2017). The expression level of SDF-1α is significantly reduced in AD patients and negatively correlated with markers of synaptic loss and microglia activation (Sanfilippo et al., 2020). A study of AD mice found that SDF-1α can facilitate bone marrow-derived microglia to migrate into the brain, leading to a reduction in Aβ accumulation by enhancing the Aβ phagocytosis (Wang et al., 2012). A decrease in SDF-1α expression is related to the excessive production of APP in transgenic mice, contributing to cognitive defects, while SDF-1α pretreatment in AD mice model was found to reduce neuronal dendritic degeneration and neuronal apoptosis (Raman et al., 2011).

Other DPP4 substrates have also been shown to be neuroprotective, and use of DPP4i can increase their expression levels. Neuropeptide-Y (NPY) is the best characterized DPP4 substrate in blood circulation. NPY and its receptors are also widely expressed in the central nerve system (CNS) showing to attenuate neuroinflammation, promote neuro-proliferation and the production of sufficient trophic support for the growth of new neurons (Duarte-Neves et al., 2016). Studies in both humans and animal models reported decreased NPY levels in hippocampus and cerebral cortex regions in AD patients (Ye et al., 2018). Overexpression of NPY via DPP4i may be protective against AD.

### 3.3 Anti-inflammation, anti-oxidation, and anti-apoptosis properties of DPP4i

In addition to the mechanisms illustrated previously, DPP4i may offer additional neurocognitive benefits through anti-inflammation, anti-oxidation, and anti-apoptosis (Figure 3) (Yin et al., 2022).

The anti-inflammatory feature of DPP4i has been documented in various neurodegenerative disorders. Studies of rat models showed that pro-inflammatory cytokines in the hippocampus, the prone factors of AD (Calsolaro and Edison, 2016), such as TNF-α, IL-6, and NF-κB, were significantly reduced after the administration of sitagliptin (El-Sahar et al., 2015; Siddiqui et al., 2021). In mice with moderate traumatic brain injury, sitagliptin was observed to exert a neuroprotective effect by increasing the expression of anti-inflammatory factor IL-10 in the cerebral cortex and striatum (Hung et al., 2020). Reducing the expression of these cytokines via DPP4i can also inhibit the expression of NF-κB and further reduce the level of BACE-1 enzyme in neurons, a key component involved in the amyloidogenic pathway to produce Aβ oligomers (Chen et al., 2012). In addition, DPP4i was shown to reduce the differentiation of macrophages into M1 phenotype, with the latter associated with neuroinflammation, and to induce differentiation of macrophages into M2 phenotype to exert neuroprotective effects (Wiciński et al., 2018).

One major cause of oxidative stress in the human body is hyperglycemia (Fiorentino et al., 2013). DPP4i has been shown to alleviate oxidative stress. An early study of rat models with lipopolysaccharide (LPS)-induced sepsis found that linagliptin can reduce LPS-induced endothelial dysfunction, reactive oxygen species (ROS) generation, the NADPH oxidase subunits expression and aortic infiltration with inflammatory cells in the vascular and cardiac tissues and blood (Kroller-Schon et al., 2012). After the treatment of sitagliptin, the levels of glutamate and nitric oxide decreased significantly in the hippocampus of ischemic rats, while the concentration of glutathione increased significantly (El-Sahar et al., 2015). Li *et al.* assessing the effect of sitagliptin combined with quercetin for the treatment of AD found that the combined administration not only significantly reduced the level of Aβ, but also enhanced the Nrf2/HO-1 pathway and improved the antioxidant activity in the brain of rat models (Li et al., 2019).

Moreover, DPP4i was found to reduce neuronal cell apoptosis and promote neurogenesis (Kornelius et al., 2015). In an experimental study of human neuronal cells, linagliptin was observed to mitigate Aβ-induced cytotoxicity by activating the AMPK-Sirt1 signaling pathways. The proportion of apoptotic cells in the total cells decreased significantly from 35% to 20% (*p* < 0.01) (Kornelius et al., 2015). Vildagliptin use was found to prevent neuronal apoptosis in hippocampus, reduce the expression of apoptosis-related proteins and increased neurotrophic factors in rat models of T2DM (Zhang et al., 2018).

### 3.4 Other mechanisms underlying the potential neuroprotective effects of DPP4i

There are other mechanisms underlying the potential neuroprotective effects of DPP4i. For example, Sakr *et al.* studied the working memory and reference memory of T2DM rats with and without sitagliptin treatment by using the hole-board memory test and isolated rat’s hypothalamus to measure levels of acetylcholine and adiponectin receptor 1 (Adipo R1) mRNA expression (Sakr, 2013). The results showed that sitagliptin treatment significantly improved working memory from 50.67% to 63.5% and reference memory from 43.33% to 69.76% in T2DM rats (both *p* < 0.0001). In addition, sitagliptin significantly increased the content of acetylcholine and the expression of Adipo R1 in the hypothalamus, providing an insight into the mechanisms underlying the neuroprotective effects of sitagliptin (Sakr, 2013). Dong *et al.* reported that sitagliptin can improve learning and memory function by enhancing synaptic plasticity through stimulating BDNF-TrkB signal transduction pathway (Dong et al., 2019). Recent studies have shown that sitagliptin improved L-methionine-induced vascular dementia and cognitive deficits through its antioxidant, anti-inflammatory, anti-apoptosis and neurotrophic effects (Khodir et al., 2022). In addition, DPP4i was found to increase serum Sirtuin 1 level in T2DM patients with AD (Kornelius et al., 2015). Sirtuin 1 is a class III histone deacetylase known for its benefit in cognitive function by enhancing synaptic plasticity, improving memory through regulation of CREB and BDNF expression, and reducing Aβ accumulation, oxidative stress, and neuronal loss (Kumar et al., 2013). Substance P, a neuropeptide widely presented in the CNS and a substrate of DPP4, was found to be protective against AD by ameliorating Aβ-induced neuronal apoptosis in the brain secondary to the stimulation of non-amyloidogenic APP processing (Severini et al., 2016). The increased bioactivity of GLP-2 by DPP4i may improve spatial working memory in juvenile diabetic rats through MEK/ERK pathway (Sasaki-Hamada et al., 2021).

## 4 Epidemiological evidence supporting the benefits of DPP4i in preventing AD

In addition to the experimental evidence presented both *in vitro* and *in vivo*, accumulative evidence from clinical studies have supported the neuroprotective effects of DPP4i in AD (Table 1). In a small-scale study of older patients with T2DM and mild cognitive impairment (n = 250), DPP4i-based glucose-lowering therapy was associated with a slower deterioration of cognitive function, mainly attentional and executive functions, compared to the sulfonylurea-based glucose-lowering therapy over a 2-year follow-up (Odds ratio: 0.88, 95% CI 0.45–0.99, *p* = 0.03) (Rizzo et al., 2014). This association appeared to be independent of its sustained hyperglycemia and glucose excursion. Another small-scale study of 253 older T2DM patients with and without AD yielded a similar conclusion that DPP4i use was associated with better cognitive performance compared with metformin (mean [SD] change in MMSE score in sitagliptin group *versus* metformin group over 6 months in patients without AD: 0.95 ± 2.17 *versus* −2.50 ± 3.03) (Isik et al., 2017). Nasir *et al.* found that, when used in combination with metformin, DPP4i was associated with better cognition, when compared with sulphonylureas, alpha glucosidase inhibitors, and thiazolidinediones (*p* < 0.05), with mean and SD in MMSE score of 29.11 ± 0.19, 24.64 ± 0.38, 25.33 ± 0.73, and 21.36 ± 1.77, respectively (Nair et al., 2019). Akimoto *et al.* conducting a regression analysis for the risk of AD on different antidiabetic drug therapies found that the risk of AD was significantly reduced by sitagliptin treatment compared with metformin monotherapy (adjusted odds ratio: 0.75; 95% confidence interval [CI]: 0.60–0.93; *p* = 0.011) (Akimoto et al., 2020).

**TABLE 1:** Clinical studies investigating the association between DPP4i use and cognitive outcomes.

Authors, year	Study population	Study design	Intervention (exposure) group	Control group	Participants number	Follow-up period	Outcomes	Results
Rizzo et al. (2014)	T2DM elderly people with mild cogni-tive impair-ment	Cohort study	DPP4i + metformin	Sulfonylurea + metformin	Intervention: 120 Control: 120	2 years	Having MMSE scores low (<25) at baseline and high (>27) after 2 y of therapy	Control vs. intervention: OR = 0.88, 95% CI: 0.45–0.99, *p* = 0.03 (which means DPP4i use was associated with cognitive improvement)
Isik et al. (2017)	T2DM elderly patients with and without AD	Cohort study	Sitagliptin	Metformin, insulin	Intervention: 101 Control: 104	6 months	Mean (SD) change in MMSE score from baseline to 6 months	In non-AD patients: Sitagliptin vs. insulin vs. metformin: 0.95 ± 2.17 vs. 0.94 ± 1.89 vs. −2.50 ± 3.03, *p* = 0.02 In AD patients: only analysis comparing sitagliptin with metformin had sufficient power, with improved cognition observed in sitagliptin users *p* = 0.047
Kim et al. (2018)	T2DM patients aged 60 years or older	Cohort study with 1:1 matching	New users of DPP4i	New users of sulfonylurea	Intervention: 7,552 Control: 7,552	Mean 3.7 years	All-cause dementia; AD, VaD	All cause dementia: RR 0.66, 95% CI 0.56–0.78; AD: RR 0.64, 95% CI 0.52–0.79; VaD: RR 0.66, 95% CI 0.38–1.14
Bohlken et al. (2018)	T2DM patients	Case-control	Ever use of DPP4i	Never use of DPP4i	Case: 8,276 Control: 8,276	5 years	All-cause dementia	RR 0.99, 95% CI 0.91–1.07
Nair et al. (2019)	T2DM patients	Cross-sectional	Metformin + DPP4i	Metformin + sulfonylureas or metformin + alphaglucosidase inhibitors or metformin + thiazolidinediones	Intervention: 151 Control 325	N/A	Mean (SD) of MMSE score	Metformin + DPP4i vs. Metformin + sulphonylureas vs. Metformin + Alpha glucosidase inhibitors vs. Metformin + thiazolidinediones: 29.11 ± 0.19 vs. 24.64 ± 0.38 vs. 25.33 ± 0.73 vs. 21.36 ± 1.77; P for metformin + DPP4i vs. each of the other three groups: <0.05
Biessels et al. (2019)	T2DM patients with cardiorenal comorbidities	Sub-study of a randomized trial	Linagliptin 5 mg daily	Placebo	Intervention: 800 Control: 745	Median 2.5 years	Accelerated cognitive decline^a^	OR 0.96, 95% CI 0.77, 1.19, *p* = 0.69
Borzi et al. (2019)	T2DM elderly people with mild cognitive impairment	Cohort study	Vildagliptin + metformin	Metformin monotherapy	Intervention: 30 Control: 30	6 months	Mean (SD) change in MMSE score from base-line to 6 months	Metformin + vildagliptin vs. Metformin at end of follow-up: 21.27 ± 1.44 vs. 19.67 ± 1.47, *p* < 0.001
Wium-Andersen et al. (2019)	T2DM patients	Case control	Ever use of DPP4i	Never use of DPP4i	Case: 11,619 Control: 46,476	From 1995 to 2012	All-cause dementia	RR 0.80, 95% CI 0.74–0.88
Kim et al. (2018)	Subjects aged 60 years or older	Cohort study	Ever use of DPP4i	Never use of DPP4i	Total: 116,139	Mean 11 years	All-cause dementia	DPP4i monotherapy - RR 0.31.95% CI (0.12–0.82); DPP4i combination therapy: RR 0.48; 95% CI 0.45–0.51
Xue et al. (2020)	T2DM elderly patients with post-stroke mild cognitive impairment	Cohort study	DPP4i	Sulfonylurea	Intervention: 30 Control: 30	6 months	Mean (SD) of MMSE and MoCA scores	After treatment –Intervention vs. control: MMSE, 26.83 ± 0.91 vs. 22.70 ± 1.80, *p* < 0.001; MoCA, 23.73 ± 2.03 vs. 22.23 ± 2.18
Akimoto et al. (2020)	T2DM patients aged 65 years or older	Cohort study	Sitagliptin	Other anti-diabetic monotherapies	Intervention: 149 Control: 6,832	Not reported	AD	Alogliptin: OR 1.27, 95%CI 0.73–2.06, *p* = 0.35; Saxagliptin: OR 0.88, 95%CI 0.52–1.39, *p* = 0.61; Sitagliptin: OR 0.75, 95%CI 0.60–0.93, *p* = 0.01; Linagliptin: OR 0.67, 95% CI 0.40–1.07, *p* = 0.11
Ates Bulut et al. (2020)	T2DM patients	Cohort study	Vildagliptin	No vildagliptin	Intervention: 43 Control: 52	6 months	Mean (SD) change in MMSE score from baseline to 6 months	Vildagliptin (+) vs. vildagliptin (−): −0.27 ± 1.07 vs. -0.20 ± 0.93, *p* = 0.50
Chen et al. (2020)	T2DM patients without dementia and aged over 50	Cohort study with propensity score matching	Use of DPP4i	Not use of DPP4i	Intervention: 2,903 Control: 11,612	Mean 7 years	All-cause dementia; AD, VaD	All cause dementia: RR 0.80, 95% CI 0.68–0.88; AD: RR 0.89, 95% CI 0.71–1.27; VaD: RR 0.58, 95% CI 0.40–0.68
Secnik et al. (2021)	T2DM patients with diagnosed dementia	Cohort study	DPP4i	No DPP4i	Intervention: 103 Control: 389	3 years	Weighted annual mean (95%CI) change in MMSE score	0.72 (0.06–1.37), *p* = 0.03
Tseng (2021)	Patients with newly diagnosed DM	Cohort study with propensity score matching	Vildagliptin	No vildagliptin	Intervention: 40,489 Control: 40,489	2 years	All-cause dementia	RR 0.83, 95% CI 0.50–1.37
Zhou et al. (2021)	Patients with T2DM	Retrospective cohort study	Ever use of DPP4i	Never use of DPP4i	Intervention: 15,409 Control: 327,017	5 years	AD	RR 0.96, 95% CI 0.90–1.02

Abbreviations: AD, Alzheimer’s disease; CI, confidence interval; DPP4i, Dipeptidyl peptidase 4 inhibitor; MMSE, Mini-Mental State Examination; N/A, not applicable; OR, odds ratio; RR, risk ratio; SD, standard deviation; T2DM, type-2 diabetes; VaD, vascular dementia.

^a^ Accelerated cognitive decline was defined as a regression-based index score ≤16th percentile on the MMSE, or a composite measure of attention and executive functioning and analyzed in participants with a baseline MMSE ≥24.

There are more clinical studies being conducted in recent years. A Korean study using health insurance claim database found that DPP4i use *versus* sulfonylurea use was associated with a 34% reduced risk of incident all-cause dementia (95% CI: 0.56–0.78; *p* < 0.001) and 36% reduced risk of AD (95% CI: 0.52–0.79; *p* < 0.001) in older adults with T2DM (Kim et al., 2018). This finding in Asian populations was agreed by another study using data of European cohort, in which DPP4i use was found to be associated with a slower cognitive decline in patients with T2DM who were recently diagnosed with dementia, when compared with no treatment use, insulin, and sulfonylureas (Secnik et al., 2021). A recent meta-analysis of observational studies published in 2021 reported that DPP4i use was associated with a significantly reduced risk of all-cause dementia (Hazard ratio [HR]: 0.65, 95% CI, 0.55–0.76) and AD (HR: 0.48, 95% CI, 0.25–0.92), when compared with no glucose-lowering treatment (Zhou et al., 2020). Another newer meta-analysis yielded a similar conclusion that DPP4i use was associated with a significantly reduced risk of all-cause dementia (Risk Ratio [RR], 0.84; 95% CI, 0.74–0.94) and vascular dementia (RR, 0.59; 95% CI, 0.47–0.75) compared with no DPP4i use (Tang et al., 2023). However, when compared with other glucose-lowering medications, the association between DPP4i and AD (RR, 0.82; 95% CI, 0.63–1.08) is not statistically significant but there is still a trend towards positive despite a high between-study heterogeneity (Tang et al., 2023).

As of now, no randomized trial has been completed yet to investigate the effect of DPP4i on prevention and treatment of AD. Clinical studies in GLP-1RAs and SGLT2is for treating AD are lesser. GLP-1RAs work by directly activating GLP-1 receptors on pancreatic islet β-cells, δ-cells, and α-cells to increase insulin release and decrease glucagon secretion (Ussher and Drucker, 2023). SGLT2i lowers blood glucose and increase glycosuria levels by preventing glucose reabsorption in kidney through inhibiting SGLT2, the primary sodium-coupled glucose transporter in renal proximal tubules (Cassis et al., 2018). Like DPP4is, both SGLT2is and GLP1-RAs have been shown in many pre-clinical studies that can confer neuroprotective benefits beyond their effects on glucose-lowering. These include ameliorating the accumulation of Aβ plaques, oxidative stress and neuroinflammation, inhibiting acetylcholinesterase activity, and reducing cerebrovascular damage and neuronal cell death (Hayden et al., 2019; Hierro-Bujalance et al., 2020; Kaneto et al., 2021; Du et al., 2022; Chen et al., 2023; Mancinetti et al., 2023; Pelle et al., 2023; Piatkowska-Chmiel et al., 2023). Given that these novel anti-diabetic medications hold great promise for reducing AD in T2DM patients, well-performed randomized trials with sufficient sample size and follow-up are urgently needed to inform evidence-based clinical practice and new therapeutic approaches for dementia and AD.

## 5 Conclusion

DPP4i, a class of novel glucose-lowering medications that has been used in recent years, holds a great promise in preventing AD development and progression. Beyond its efficacy in glucose control and improvement of neuronal insulin resistance, DPP4i may provide neurocognitive benefits by directly reducing Aβ deposition and tau hyperphosphorylation. DPP4i can also increase the expression and bioavailability of neuroprotective DPP4 substrates such as GLP-1, GIP, SDF-1α, and NPY. Furthermore, a growing body of evidence substantiates the diverse biological functions of DPP4i in the brain, including anti-inflammatory, anti-oxidative, and anti-apoptotic effects, along with the promotion of neurogenesis. These properties collectively contribute to the amelioration of neurodegeneration and provide direct protection against Aβ-induced neurotoxicity. Randomized trials are needed to provide a definitive conclusion on the effect of DPP4i on AD prevention and treatment in T2DM patients and individuals at high risk of AD.

## Figures and Tables

**Table:** 

**AD**	Alzheimer’s disease
**Adipo R1**	Adiponectin receptor 1
**AGE**	Advanced glucation end product
**AMPK-Sirt1**	Adenosine monophosphate activated protein kinase- sirtuin 1
**APP**	Amyloid precursor protein
**Aβ**	Amyloid- beta
**BACE-1**	Beta-site APP-cleaving enzyme 1
**BBB**	Blood-brain barrier
**BDNF**	Brain derived neurotrophic factor
**BDNF-TrkB**	Brain derived neurotrophic factor- Tyrosine receptor kinase B
**cAMP-/PKA/CREB**	cAMP-dependent protein kinase A/cAMP-response element binding protein
**CI**	Confidence interval
**CNS**	Central nerve system
**CREB**	cAMP-response element binding protein
**DDP4i**	Dipeptidyl peptidase 4 inhibitor
**ERK**	Extracellular signal regulated kinase
**GC**	Glutamyl cyclase
**GIP**	Glucose-dependent insulinotropic peptide
**GLP-1**	Glucagon-like peptide-1
**GLP-1R**	GLP-1 receptors
**GLP-1RAs**	Glucagon-like peptide-1 receptor agonists
**GSK-3**	Glycogen synthase kinase- 3
**GSK3β**	Glycogen synthase kinase- 3β
**HR**	Hazard ratio
**IL-10**	Interleukin-10
**IL-6**	Interleukin- 6
**IRS**	Insulin receptor substrate
**JNK**	c-Jun N-terminal kinase
**LPS**	Lipopolysaccharide
**MAPK**	Mitogen activated protein kinase
**MEK/ERK**	Mitogen-activated protein kinase/Extracellular signal regulated kinase
**MMP**	Matrix metalloproteinase
**MoCA**	Montreal cognitive assessment
**NADPH**	Nicotinamide adenine dinucleotide phosphate
**NF-κB**	Nuclear factor kappa-B
**NPY**	Neuropeptide-Y
**Nrf2/HO-1**	Nuclear factor- E2- related factor 2/Heme Oxygenase- 1
**p38**	Protein 38
**pE-Aβ3-40/42**	Pyroglutamate- amyloid beta3- 40/42
**PI3-K/Akt**	Phophatidylinositol-3 kinase/protein kinase B
**Pro 3 –GIP**	Antagonist of GIP
**ROS**	Reactive oxygen species
**SDF-1α**	stromal-derived factor-1α
**sDPP4**	Soluble Dipeptidyl peptidase 4
**SGLT2i**	Sodium-glucose cotransporter-2 inhibitors
**T2DM**	Type 2 diabetes mellitus
**TNF-α**	Tumor necrosis factor-α
